# Study on Influencing Factors of Carbon Emissions from Energy Consumption of Shandong Province of China from 1995 to 2012

**DOI:** 10.1155/2014/684796

**Published:** 2014-04-07

**Authors:** Jiekun Song, Qing Song, Dong Zhang, Youyou Lu, Long Luan

**Affiliations:** School of Economics and Management, China University of Petroleum, Qingdao 266580, China

## Abstract

Carbon emissions from energy consumption of Shandong province from 1995 to 2012 are calculated. Three zero-residual decomposition models (LMDI, MRCI and Shapley value models) are introduced for decomposing carbon emissions. Based on the results, Kendall coordination coefficient method is employed for testing their compatibility, and an optimal weighted combination decomposition model is constructed for improving the objectivity of decomposition. STIRPAT model is applied to evaluate the impact of each factor on carbon emissions. The results show that, using 1995 as the base year, the cumulative effects of population, per capita GDP, energy consumption intensity, and energy consumption structure of Shandong province in 2012 are positive, while the cumulative effect of industrial structure is negative. Per capita GDP is the largest driver of the increasing carbon emissions and has a great impact on carbon emissions; energy consumption intensity is a weak driver and has certain impact on carbon emissions; population plays a weak driving role, but it has the most significant impact on carbon emissions; energy consumption structure is a weak driver of the increasing carbon emissions and has a weak impact on carbon emissions; industrial structure has played a weak inhibitory role, and its impact on carbon emissions is great.

## 1. Introduction

In recent years, Shandong province of China has achieved remarkable performance in economic development. However, Shandong has also been facing problems with excessive energy consumption and increasing carbon emissions. This study aims to obtain the characteristics of temporal variation of carbon emissions from energy consumption and analyze the influencing factors of carbon emissions, which can help Shandong province work out scientific and reasonable reduction strategies of carbon emissions. The study is significant for promoting economic and social sustainable development of Shandong province.

Factors decomposition has been widely used to study the driving forces of an aggregate indicator's change over time. Energy and environment researchers have put forward many decomposition models. According to different classification standards, decomposition models can be divided into various types. According to equality expression of carbon emissions, decomposition models can be categorized into additive decomposition and multiplicative decomposition models; according to the decomposition with residual or not, decomposition models can be categorized into residual decomposition and zero-residual decomposition models; according to the principle of method, decomposition models can be categorized into index decomposition and structural decomposition models [1].

For logarithmic mean weight Divisia index (LMDI) model is simple and its result does not include unexplained residuals [2–5], it has become one of the most widely used decomposition models. By using LMDI, Hatzigeorgiou et al. [6] decomposed carbon emissions of Greece. Wei et al. [7], Song and Lu [8], Zhang et al. [9], and Wang et al. [10] used LMDI to decompose carbon emissions of China into four factors including output scale, energy structure, emission intensity, and energy intensity. Zhu et al. [11] decomposed it into five factors including population scale, economy yield, industrial structure, energy intensity, and energy structure. Zhang and Ren [12] and Song [13] used LMDI to decompose carbon emissions of Shandong province into the same five factors. By using LMDI, Guo et al. [14] decomposed carbon emissions of Shanghai city into four factors including population, per capita GDP, energy intensity, and energy structure. Liu et al. [15, 16] decomposed carbon emissions of Fujian province and Beijing city into four factors including energy structure, emission intensity, energy intensity, and economy development. Zhao and Long [17] decomposed carbon emissions of Jiangsu province from the angle of three industries by using LMDI. Besides LMDI, mean-rate-of-change index model [18], refined Laspeyres index model [19], and Shapley value model [20] are zero-residual decomposition models. Ang et al. [21] proved that Shapley value decomposition is in fact the same as the refined Laspeyres index decomposition mathematically. Since the above models are suitable for the decomposition of carbon emissions and have no unexplained residuals, whose result is most objective and credible, or can we combine them to get the better result? This paper proposes a combination method and applies it to decompose carbon emissions from energy consumption of Shandong province.

This paper is organized as follows.  Section 2 gives the situations of economy and energy consumption and calculates carbon emissions of Shandong province from 1995 to 2012.  Section 3 constructs factors decomposition models of carbon emissions from energy consumption including LMDI, Shapley value, mean-rate-of-change index (MRCI), and combination models.  Section 4 decomposes carbon emissions from energy consumption of Shandong province into different factors by applying the constructed models.  Section 5 constructs the STIRPAT model to analyze the impacts of population, affluence, and technology on carbon emissions of Shandong province. Conclusions and implications are presented in Section 6.

## 2. Economy, Energy Consumption, and Carbon Emissions of Shandong Province 

### 2.1. Economic Development of Shandong Province

**Figure 1:**
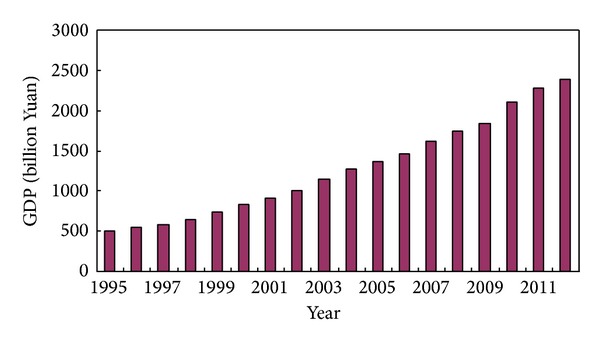
GDP and growth rate of Shandong province from 1995 to 2012. Source: Shandong Statistics Yearbook 2013.

**Figure 2:**
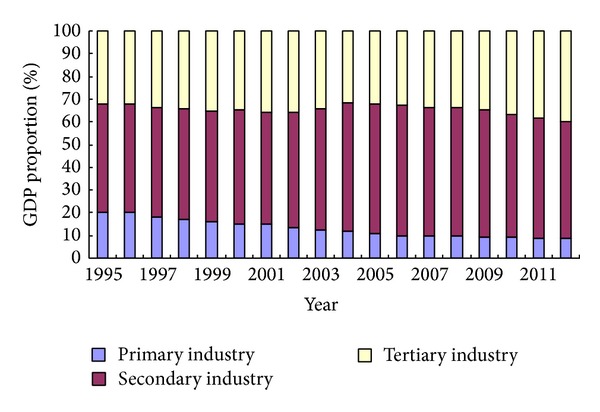
Industrial structure of Shandong province from 1995 to 2012. Source: Shandong Statistics Yearbook 2013.

GDP of Shandong province from 1995 to 2012 maintained a rapid growth trend (see Figure 1). According to constant price of 1995, GDP in 2012 was 23880.334 billion Yuan, which was 4.82 times of GDP in 1995. The average annual GDP growth rate of Shandong province was 9.7% from 1995 to 2012. Meanwhile, industrial structure of Shandong province from 1995 to 2009 was continually optimized, which changed from 20.4 : 47.6 : 32 in 1995 into 8.6 : 51.4 : 40.0 in 2012 (see Figure 2).

### 2.2. Energy Consumption of Shandong Province

**Figure 3:**
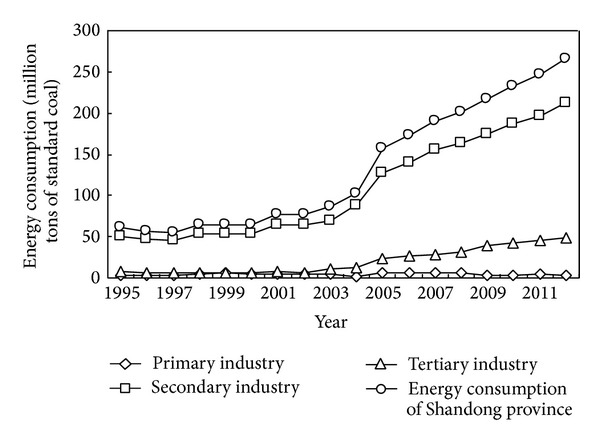
Energy consumption of Shandong province from 1995 to 2012. Source: Shandong Statistics Yearbook 1996–2013.

Energy consumption of Shandong province from 1995 to 2012 is shown in Figure 3, and we can see that total energy consumption, energy consumption of secondary and tertiary industries of Shandong province, all showed the upward trend, especially from 2004 to 2012. Energy consumption of the secondary industry accounted for the largest proportion among three industries, which was always about 80%. The lowest point of the proportion of the tertiary industry was 8.8% in 1999, and the highest point of it was 18.62% in 2012. Contradicted with the proportion of the tertiary industry, the highest point of the proportion of the primary industry was 8.51% in 1999, and the lowest point of it was 1.29% in 2012.

**Figure 4:**
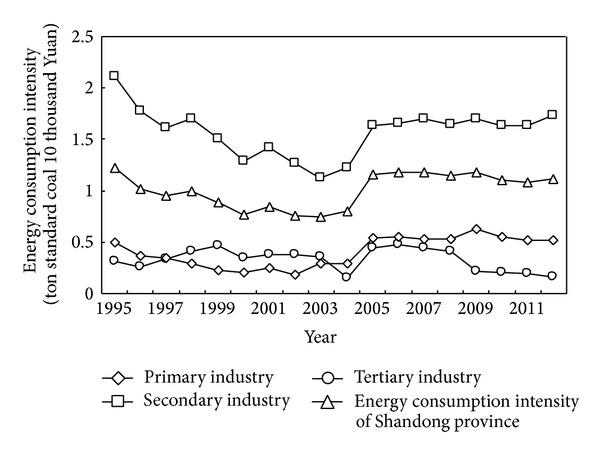
Energy consumption intensity of Shandong province from 1995 to 2012.

Energy consumption intensity of Shandong province from 1995 to 2012 is shown in Figure 4. Energy consumption intensity of the secondary industry was the highest among three industries, and it was always more than 1.1 ton standard coal per 10 thousand Yuan, which was much higher than those of the primary and tertiary industries. Energy consumption intensity of the whole province and three industries fluctuated before 2005, but those of the whole province, secondary and tertiary industries all basically showed steady trend from 2005 to 2012, and that of the primary industry showed downward trend from 2005 to 2012.

**Figure 5:**
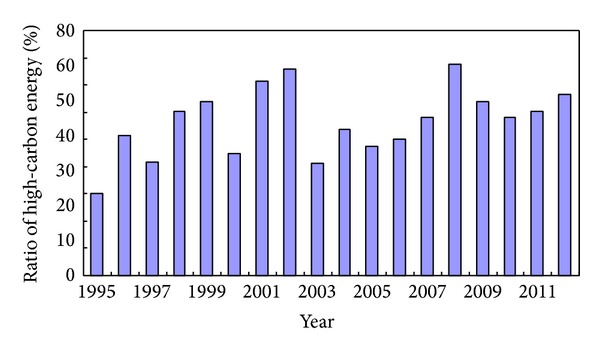
Ratio of high-carbon energy of Shandong province from 1995 to 2012. Source: Shandong Statistics Yearbook 1996–2013.

Figure 5 shows the ratio of high-carbon energy in terminal energy consumption of Shandong province during 1995–2012, where high-carbon energy include raw coal, cleaned coal, other washed coal, briquettes, coke, heat and electricity, and their carbon emissions coefficients are all more than 0.75 kg/kg standard coal. We can see that the ratio fluctuated over the eighteen years, but it was always maintained higher than 68%.

### 2.3. Carbon Emissions from Energy Consumption of Shandong Province

The method which uses carbon emissions coefficient is currently the most widely used method for calculating carbon emissions. The calculation formula is

(1) $$\begin{array}{c} C=\overset{3}{\underset{i=1}{\sum }}\overset{19}{\underset{j=1}{\sum }}C_{ij}=\overset{3}{\underset{i=1}{\sum }}\overset{19}{\underset{j=1}{\sum }}E_{ij}\times r_{j}, \end{array}$$

where *C* is carbon emissions from energy consumption, *C*_*ij*_ and *E*_*ij*_ are carbon emissions and energy consumption from the* j*th energy in the *i*th industry, respectively, and *r*_*j*_ is the carbon emissions coefficient of the* j*th energy.

**Figure 6:**
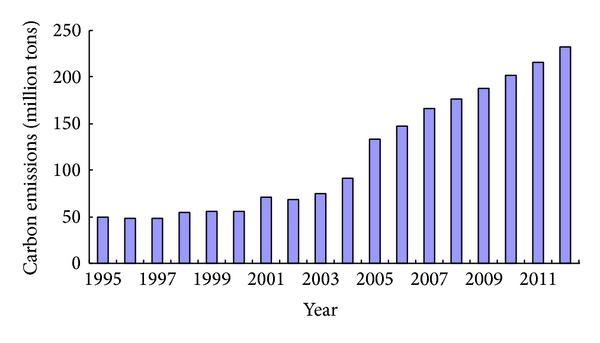
Carbon emissions from energy consumption of Shandong province from 1995 to 2012.

We have calculated the carbon emissions coefficients of 17 kinds of energy of Shandong province [13]. By using energy balance table of China, we calculate that the carbon emissions coefficients of heat energy and electricity are 0.8760 and 1.6672 kg/kg standard coal, respectively. Applying the statistical data of energy consumption of Shandong province from 1995 to 2012, the calculated carbon emissions from energy consumption of Shandong province are shown in Figure 6. The overall carbon emissions from energy consumption of Shandong province show an increasing trend. Particularly, carbon emissions have increased significantly from 68.3809 million tons in 2002 to 232.4380 million ton in 2012, and the annual average growth rate was 13.02%.

## 3. Factors Decomposition Model of Carbon Emissions from Energy Consumption 

### 3.1. LMDI Model

LMDI has become the most commonly used decomposition method of carbon emission. Generally, the factors include GDP, population, per capita GDP, industrial structure, energy consumption structure, energy consumption intensity, carbon emission coefficients, and so on. According to the combination form of the above factors, decomposition models can be classified into three factors decomposition, four factors decomposition, five factors decomposition, six factors decomposition, and so forth. For example, an equation of carbon emissions with six factors is as follows:

(2) C=∑i=13∑j=1nP×GP×GiG×EiGi×EijEi×CijEij=∑i=13∑j=1nP×a×si×ei×mij×dij,

where *P* is population, *G* is GDP, *G*_*i*_ is GDP of the *i*th industry, *E*_*i*_ is energy consumption of the *i*th industry, *E*_*ij*_ is consumption of the *j*th energy in the *i*th industry, *C*_*ij*_ is the carbon emissions from the *j*th energy in the *i*th industry, *a* is per capita GDP, *s*_*i*_ is GDP proportion of the *i*th industry, *e*_*i*_ is energy consumption intensity of the *i*th industry, *m*_*ij*_ is consumption proportion of the *j*th energy in the *i*th industry, and *d*_*ij*_ is carbon emission coefficient of the *j*th energy in the *i*th industry.

To be more general, we decompose carbon emissions into *l*_1_ + *l*_2_ + *l*_3_ kinds of factors, and the carbon emissions equation is

(3) C=∑i=1m∑j=1n[(x1×⋯×xl1)×(y1,i×⋯×yl2,i)×(z1,ij×⋯×zl3,ij)].

According to LMDI model, we firstly calculate the lowest layer of carbon emissions:

(4) Cij=(x1×⋯×xl1)×(y1,i×⋯×yl2,i) ×(z1,ij×⋯×zl3,ij).

Then the carbon emissions change from the (*t* − 1)th year to the* t*th year is:

(5) $$\begin{array}{c} \Delta C=C^{t}-C^{t-1}=\overset{l_{1}}{\underset{k=1}{\sum }}\Delta C_{x_{k}}+\overset{l_{2}}{\underset{k=1}{\sum }}\Delta C_{y_{k}}+\overset{l_{3}}{\underset{k=1}{\sum }}\Delta C_{z_{k}}. \end{array}$$

Let *u* denote any of decomposition factors  *x*_1_,…, *x*_*l*_1__, *y*_1,*i*_,…, *y*_*l*_2_,*i*_, *z*_1,*ij*_,…, *z*_*l*_3_,*ij*_, then the effect of factor *u* is

(6) $$\begin{array}{c} \Delta C_{u}=\overset{m}{\underset{i=1}{\sum }}\overset{n}{\underset{j=1}{\sum }}\left[L\left(C_{ij}^{t-1},C_{ij}^{t}\right)\ln\left(\frac{u^{t}}{u^{t-1}}\right)\right], \end{array}$$

where

(7) $$\begin{array}{c} L\left(C_{ij}^{t-1},C_{ij}^{t}\right)=\{\begin{array}{cc} \frac{C_{ij}^{t}-C_{ij}^{t-1}}{\ln\left(C_{ij}^{t}/C_{ij}^{t-1}\right)}, & C_{ij}^{t}\neq C_{ij}^{t-1}\\ C_{ij}^{t}, & C_{ij}^{t}=C_{ij}^{t-1}. \end{array} \end{array}$$

Because *C*_*ij*_^*t*^ and *u* ^*t*^ may be 0, there are four exceptional cases listed in Table 1.

### 3.2. Shapley Value Model

Albrecht et al. [20] put forward Shapley value decomposition model based on the Kaya equation, and we extend their conclusion to the general case shown in formula (3). Let *u* denote any factor, *N*_*ij*_ = {*x*_1_,…, *x*_*l*_1__, *y*_1,*i*_,…, *y*_*l*_2_,*i*_, *z*_1,*ij*_,…, *z*_*l*_3_,*ij*_} denote players set, and union *U*⊆*N*_*ij*_,  *U* ≠ Φ, then we define the characteristic function as follows:

(8) $$\begin{array}{c} v\left(U\right)=\underset{d\in U}{\prod }d^{t}*\underset{\overset{-}{d}\in N_{ij}\setminus U}{\prod }{\overset{-}{d}}^{t-1}Q. \end{array}$$

The effect of factor *u* is

(9) ΔCu=∑i=1m∑j=1nΔCu,ij,ΔCu,ij∑r=1l1+l2+l3(r−1)!(l1+l2+l3−r)!(l1+l2+l3)! ×∑U:u∈U|U|=r[v(U)−v(U∖{u})],

where |*U*| is the base of union *U*, that is, the number of players in it.

### 3.3. MRCI Model

MRCI model uses the proportion of each factor's average change rate as its weight, and the effect of factor *u* is

(10) $$\begin{array}{c} \Delta C_{u}=\overset{m}{\underset{i=1}{\sum }}\overset{n}{\underset{j=1}{\sum }}\left[\frac{C_{ij}^{t}-C_{ij}^{t-1}}{A_{ij}}\times \frac{u^{t}-u^{t-1}}{\left(u^{t}+u^{t-1}\right)/2}\right], \end{array}$$

where *u* denotes any factor; *A*_*ij*_ denotes the sum of all the factors' average change rate:

(11) Aij=∑k=1l1xkt−xkt−1(xkt+xkt−1)/2+∑k=1l2yk,it−yk,it−1(yk,it+yk,it−1)/2 +∑k=1l3zk,ijt−zk,ijt−1(zk,ijt+zk,ijt−1)/2.

If both *u* ^*t*^ and *u* ^*t*−1^ are 0, we define the effect of factor *u* is 0.

### 3.4. Effect Contribution

In order to reflect the effect contribution of each factor, we define the effect contribution of factor *u* as follows:

(12) $$\begin{array}{c} \eta_{u}=\mathrm{sgn}\left(\Delta C\right)\frac{\Delta C_{u}}{\Delta C}, \end{array}$$

where sgn(Δ*C*) indicates the change direction of carbon emissions; that is, if carbon emissions increase, it is positive; otherwise, it is negative:

(13) $$\begin{array}{c} \mathrm{sgn}\left(\Delta C\right)=\{\begin{array}{cc} +1, & \Delta C>0\\ -1, & \Delta C<0 \end{array} \end{array}$$

Thus, if *η*_*u*_ > 0, we think that the factor *u* promotes the increase of carbon emissions; otherwise, it inhibits the increase of carbon emissions.

### 3.5. Combination Decomposition Model

Each decomposition model has its own theoretical basis, and the above three models are usually applicable to the decomposition of carbon emissions. Because there may be great difference between the results of different models, we use Kendall coordination coefficient method to test their compatibility [22, 23]. Let Δ*C*_*kr*_^*t*^ and *η*_*kr*_^*t*^ denote the *r*th factor's effect and effect contribution in the *t*th year in the *k*th model, respectively; *r* = 1, …, *l*_1_ + *l*_2_ + *l*_3_; *k* = 1, 2, 3; *t* = 1, 2, …, *T*, then we sort all the values of *η*_*kr*_^*t*^ in the* k*th model from big to small, and the smaller value means the stronger inhibitory effect on the increase of carbon emissions. Suppose the sort of *η*_*kr*_^*t*^ is *p*_*kr*_^*t*^, *p*_*kr*_^*t*^ ∈ {1,2,…, *T*(*l*_1_ + *l*_2_ + *l*_3_)}, and the sort result should be consistent in different models. We can calculate the statistics index:

(14) $$\begin{array}{c} \chi^{2}=b\left(h-1\right)W, \end{array}$$

where *b* is the number of single decomposition models, *b* = 3; *h* is the number of sorted values, *h* = *T*(*l*_1_ + *l*_2_ + *l*_3_); *W* is coordination coefficient used to identify the difference between the actual sort results of different models and the most possible sort results in theory. The formula of *W* is as follows:

(15) $$\begin{array}{c} W=\frac{12\sum_{t=1}^{T}\sum_{r=1}^{l_{1}+l_{2}+l_{3}}{\left(p_{r}^{t}\right)}^{2}-3b^{2}h{\left(h+1\right)}^{2}}{b^{2}h\left(h^{2}-1\right)},\\ p_{r}^{t}=\overset{b}{\underset{k=1}{\sum }}p_{kr}^{t}. \end{array}$$

Generally, *χ* ^2^ approximately obeys *χ*_*α*_^2^(*h* − 1), where  *α*  is the given significance level. If *χ* ^2^ ≥ *χ*_*α*_^2^(*h* − 1), it indicates that the *b* kinds of models are compatible, otherwise incompatible. For incompatible case, we should eliminate some model one by one until all the rest models pass compatibility test.

Suppose there are *z* kinds of single decomposition models in compatible models set, and in order to take full advantage of their inherent information, we apply the optimal weighted combination model to combine all the compatible single models. Let *w*_*k*_ be the weight of the* k*th model, then the combination value of the* r*th factor's effect in the* t*th year is

(16) $$\begin{array}{c} \Delta C_{r}^{t}=\overset{z}{\underset{k=1}{\sum }}w_{k}\Delta C_{kr}^{t},\;r=1,\ldots ,l_{1}+l_{2}+l_{3};\;\;t=1,\ldots ,T. \end{array}$$

The objective is to minimize the square sum of deviations between all the factor's combination values in every year and their corresponding effect values in every single model, and we construct the optimal weighted combination decomposition model as follows:

(17) min⁡ ∑k=1z∑t=1T∑r=1l1+l2+l3(ΔCrt−ΔCkrt)2s.t.  ∑k=1zwk=1, wk≥0,  k=1,…,z.

Solve the above nonlinear programming model to get all the single decomposition models' weights, and input them into formula (16), then we can obtain all the factors' combination effects.

## 4. Factors Decomposition of Carbon Emissions from Energy Consumption of Shandong Province

We decompose carbon emissions from energy consumption of Shandong province into six factors, which can be seen in formula (2). We define carbon emissions increment from energy consumption as the total effect and denote it as Δ*C*, which is made up of six terms of effects, that is, population effect Δ*C*_*p*_, per capita GDP effect Δ*C*_*a*_, industrial structure effect Δ*C*_*s*_, energy consumption intensity effect Δ*C*_*e*_, energy consumption structure effect Δ*C*_*m*_, and carbon emissions coefficient effect Δ*C*_*d*_. Carbon emissions coefficient of each energy can be considered constant each year, and Δ*C*_*d*_ equals 0 [24, 25]:

(18) $$\begin{array}{c} \Delta C=\Delta C_{p}+\Delta C_{a}+\Delta C_{s}+\Delta C_{e}+\Delta C_{m}+\Delta C_{d}. \end{array}$$

LMDI model, Shapley value model, and MRCI model are used to calculate the effect of each factor, respectively, and the results are shown in Tables 2, 3, and 4.

Applying Kendall coordination coefficient method to test three models' compatibility, we get that the coordination coefficient *W* equals 0.99986, and the statistics index  *χ* ^2^  equals 251.9442. Give the significance level *α* = 0.01, 251.9442 > *χ*_0.01_^2^(84) = 117.0565, so the three models pass compatibility test. Based on the results of three models, we construct and solve the optimal weighted combination decomposition models. The calculation results are that the weights of three models are equal to 0.333339, 0.333342 and 0.333319 respectively. Inputting the above results into formula (16), we get the combination result shown in Table 5.

From the decomposition results in Table 5, we know that, using year 1995 as the base year, the cumulative effects of population, per capita GDP, energy consumption intensity, and energy consumption structure of Shandong province in 2012 were positive, but the cumulative effect of industrial structure was negative. It shows that, population, per capita GDP, and the changes in energy consumption intensity and energy consumption structure have positive impacts on the increase of carbon emissions from energy consumption, while the change of industrial structure has certain negative impacts on it. On the one hand, the cumulative effect of per capita GDP has the largest contribution, that is, per capita GDP is the most positive influence factor for the increasing carbon emissions from 1995 to 2012, while the positive effects of energy consumption intensity, population, and energy consumption structure are relatively negligible; on the other hand, industrial structure is a negative impact factor for the increasing carbon emissions.

Population effect was always positive from 1996 to 2012, but it was relatively small and its contribution is relatively weak among the five factors. It indicates that population growth is a weak drive factor for the increasing carbon emissions. Per capita GDP effect is always positive, and its contribution is the largest, which manifests that per capita GDP is the most important drive factor.

Industrial structure effect has a certain fluctuation. It was negative in 1996, 1999, 2001, and 2007–2012, and positive in the rest of the years. The trend is closely related to the industrial restructuring of Shandong province over the fifteen years. When the effect was negative in one year, GDP proportion of the secondary industry was less than in the previous year, meanwhile, GDP proportions of the tertiary industry was greater than in the previous year. Thus, reducing GDP proportion of the secondary industry and increasing GDP proportion of the tertiary industry can help to inhibit the growth trend of carbon emissions. The absolute values of industrial structure effect contribution are relatively small in the various factors, indicating that the industrial structure of Shandong province can be further optimized in order to expand its inhibitory effect on the increasing carbon emissions.

Energy consumption intensity effect fluctuated from 1996 to 2012. It was negative in 1996, 1997, 1999, 2000, 2002, 2003, 2008, 2010, and 2011, and positive in the rest of the years. The trend agrees with energy consumption intensity of Shandong province and that of the secondary industry. In the nine years when the effect was negative, energy consumption intensity of total province and that of the secondary industry were lower than previous year. In the rest of the years, they were higher than previous year. The absolute value of the of energy consumption intensity effect contribution was relatively higher among the five factors, which indicates that energy consumption intensity has a strong impact on the changes of carbon emissions from energy consumption, and it is an important inhibition factor of increasing carbon emissions.

Energy consumption structure effect shows the fluctuation trend. It was negative in 1998, 2002, 2003, 2005, 2009, and 2010, and positive in the rest years. The trend is basically consistent with the variation trend of the proportion of high-carbon energy. When energy consumption structure effect was negative in one year, the ratio of high-carbon energy consumption decreased than in the previous year, while it rose in the rest years. Thus, reducing the proportion of high-carbon energy or increasing the proportion of low-carbon energy can help to curb increasing carbon emissions. Energy consumption structure effect contribution is relatively weak in the five factors, which indicates that energy consumption structure of Shandong province has a large optimization space in helping to curb the fast growth of carbon emissions.

## 5. STIRPAT Model of Carbon Emissions from Energy Consumption of Shandong Province

The above results show that population, per capita GDP, industrial structure, energy consumption intensity, and energy consumption structure are the important impact factors of carbon emissions change of Shandong province. But how their changes affect the carbon emissions change? We use STIRPAT (Stochastic impacts by regression on population, affluence, and technology) model [26] to analyze the problem. The STIRPAT model transforms the IPAT model [27] into a stochastic model, and its expression is

(19) $$\begin{array}{c} I=aP^{b}A^{c}T^{d}\varepsilon . \end{array}$$

Take its logarithm form, and it can be changed into formula (20):

(20) $$\begin{array}{c} \ln I_{t}=a+b\ln P_{t}+c\ln A_{t}+d\ln T_{t}+\varepsilon_{t}, \end{array}$$

where* I*,* P*,* A*,* T* are environment pressure, population, affluence, and technology, respectively;**ε** is systematic error. The factors* P*,* A,* and *T* can be expanded [7, 26, 28, 29]. For facilitating the problem analysis, we introduce industrial structure and energy consumption structure into the model. The model (16) is changed into the following form:

(21) $$\begin{array}{c} \ln C_{t}=a+b\ln P_{t}+c\ln A_{t}+d\ln S_{t}+e\ln E_{t}+f\ln H_{t}+\varepsilon_{t}, \end{array}$$

where *C* is carbon emissions,* P* is population,* A* is per capita GDP,* S* is GDP proportion of the secondary industry,* E* is energy consumption intensity, and* H* is the ratio of high-carbon energy in terminal energy consumption.

We conduct bivariate correlation analysis on the carbon emissions and five impact factors and get the coefficients as shown in Table 6. It can be seen that there are significant correlations among per capita GDP, population, GDP proportion of the secondary industry, and carbon emissions. We also conduct collinearity diagnosis and get that the VIF (variance inflation factor) values of population, per capita GDP, GDP proportion of the secondary industry, energy consumption intensity, and the ratio of high-carbon energy are 174.489, 157.019, 3.239, 1.763 and 1.533, respectively. For the VIF values of population and per capita GDP are greater than 10, there are serious multicollinearity problems among the data.

Because least squares regression will lead to larger standard errors of regression coefficients, wider confidence intervals, and lower stability in parametric estimation [30], we apply partial least squares regression method to construct the regression equation of carbon emissions and its impact factors. The cross validation values of the first and second components are 0.973 and 0.521, respectively. The cumulative cross validation value of the two components is 0.987 and achieves high accuracy. The partial least squares regression equation is

(22) ln⁡C=−59.1782+6.19856ln⁡P+0.421887ln⁡A +1.49015ln⁡S+0.972377ln⁡E+0.894175ln⁡H.

**Figure 7:**
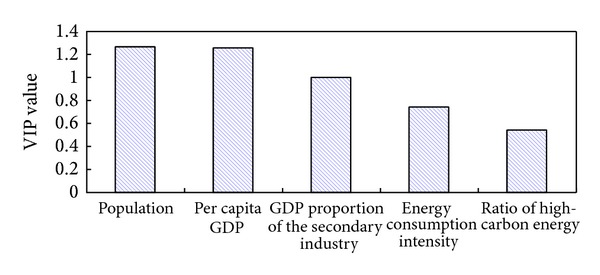
VIP values of different factors of carbon emissions from energy consumption of Shandong province.

In order to reflect the impact of each factor on carbon emissions more accurately, we calculate the VIP (variable importance in projection) value of each factor [7, 31].  Figure 7 shows that all the five factors have certain explanation roles on carbon emissions, and the rank order (high to low) is population, per capita GDP, GDP proportion of the secondary industry, energy consumption intensity, and the ratio of high-carbon energy.

## 6. Conclusions

This paper analyzes the situations of economy, energy consumption, and carbon emissions from energy consumption of Shandong province from 1995 to 2012. We construct LMDI, MRCI, and Shapley value decomposition models to decompose carbon emissions. To make full use of the underlying information in different models, the paper proposes an integrated method. We apply Kendall coordination coefficient method to perform compatibility test on the model results and output compatible model set. Based on the results of compatible models, we construct an optimal weighted combination model. We decompose carbon emissions from energy consumption of Shandong province into five factors including population, per capita GDP, industrial structure, energy consumption intensity, and energy consumption structure. We also construct the STIRPAT model of carbon emissions to reflect the impact of each factor on carbon emissions. The conclusions are as follows.

- Using 1995 as the base year, the cumulative effects of population, per capita GDP, energy consumption intensity, and energy consumption structure of Shandong province in 2012 are positive, while the cumulative effect of industrial structure is negative.
- Per capita GDP is the largest driver of the increasing carbon emissions and has a great impact on carbon emissions. Therefore, the GDP growth target of Shandong province should be appropriately reduced, so that the trend of rapid growth in carbon emissions would slow down.
- Energy consumption intensity is a weak driver of the increasing carbon emissions and has certain impact on carbon emissions. Shandong province should implement the target responsibility system in the future, and take various measures to strengthen the energy conservation in order to improve energy efficiency.
- Population affects carbon emissions with weak drive, but it has the most significant impact on carbon emissions. Shandong province should continue to control the growth of its population and, at the same time, try to improve the life quality of the population, so as to reduce the effect of consumer behavior on carbon emissions caused by the increase of population.
- Energy consumption structure is a weak drive factor of the increasing carbon emissions and has certain impact on carbon emissions. Shandong province should continue to reduce the proportion of high-carbon energy including coal and coke in the future, develop and utilize renewable low-carbon energy to achieve continuous optimization of energy consumption structure.
- Industrial structure is an inhibitory factor of the increasing carbon emissions, and the increasing proportion of tertiary industry has shown its inhibitory effect on carbon emissions since 2007. Shandong province should continue to reduce the proportion of secondary industry in the future, develop tertiary industry actively, thus to optimize industrial structure to further control the carbon emissions in the future.

## Figures and Tables

**Table 1:** Exceptional cases in LMDI model.

Case	*C*_*ij*_^*t*^	*C*_*ij*_^*t*−1^	*u* ^*t*^	*u* ^*t*−1^	*L*(*C*_*ij*_^*t*−1^, *C*_*ij*_^*t*^)ln⁡(*u* ^*t*^/*u* ^*t*−1^)	Variables represented by *u*
1	0	+	+	+	0	*x*_1_,…, *x*_*l*_1__, *y*_1,*i*_,… or *y*_*l*_2_,*i*_
2	+	0	+	+	0	*x*_1_,…, *x*_*l*_1__, *y*_1,*i*_,… or *y*_*l*_2_,*i*_
3	0	+	0	+	−*C*_*ij*_^*t*−1^	*z*_1,*ij*_,… or *z*_*l*_3_,*ij*_
4	+	0	+	0	*C*_*ij*_^*t*^	*z*_1,*ij*_,… or *z*_*l*_3_,*ij*_

**Table 2:** Effects of different factors of carbon emissions from energy consumption of Shandong province from 1996 to 2012 by LMDI model.

Year	Population	Per capita GDP	Industrial structure	Energy consumption intensity	Energy consumption structure
1996	18.484 (0.1026)	539.4969 (2.9942)	−16.9153 (−0.0939)	−906.1931 (−5.0293)	184.9446 (1.0264)
1997	25.7656 (2.1096)	201.6262 (16.5082)	58.8384 (4.8174)	−363.6212 (−29.7716)	89.6047 (7.3364)
1998	30.6057 (0.0499)	491.1051 (0.8007)	24.0681 (0.0392)	216.4525 (0.3529)	−148.8698 (−0.2427)
1999	28.0151 (0.1783)	669.2283 (4.2601)	−4.997 (−0.0318)	−623.2269 (−3.9672)	88.0744 (0.5606)
2000	70.4511 (11.1156)	637.9248 (100.65)	93.1789 (14.7015)	−888.0264 (−140.1104)	80.1336 (12.6433)
2001	30.7228 (0.0204)	515.0489 (0.3419)	−40.0778 (−0.0266)	638.2254 (0.4236)	362.7286 (0.2408)
2002	31.4352 (0.1178)	719.2631 (2.6962)	74.5668 (0.2795)	−843.8606 (−3.1632)	−248.1757 (−0.9303)
2003	33.2254 (0.0506)	848.1445 (1.2913)	286.4743 (0.4361)	−405.5063 (−0.6174)	−105.5052 (−0.1606)
2004	49.2538 (0.0298)	823.5073 (0.4984)	278.5528 (0.1686)	357.1877 (0.2162)	143.9506 (0.0871)
2005	80.8363 (0.0195)	620.4145 (0.1495)	89.8171 (0.0216)	3784.315 (0.9121)	−426.3324 (−0.1028)
2006	91.8198 (0.0624)	967.7844 (0.6573)	59.7795 (0.0406)	223.4953 (0.1518)	129.4246 (0.0879)
2007	96.6445 (0.0533)	1365.7217 (0.7531)	−102.5215 (−0.0565)	175.3475 (0.0967)	278.2693 (0.1534)
2008	90.3652 (0.0845)	1320.6287 (1.235)	−3.354 (−0.0031)	−469.1217 (−0.4387)	130.7782 (0.1223)
2009	101.3911 (0.0859)	731.2231 (0.6198)	−198.8661 (−0.1686)	644.2966 (0.5461)	−98.2134 (−0.0832)
2010	222.769 (0.1623)	2495.2563 (1.8182)	−316.2366 (−0.2304)	−1023.5432 (−0.7458)	−5.8552 (−0.0043)
2011	125.7942 (0.0931)	1434.3656 (1.0612)	−293.8969 (−0.2174)	−163.0331 (−0.1206)	248.3743 (0.1838)
2012	110.8637 (0.0657)	1008.9818 (0.5976)	−395.5105 (−0.2342)	961.0035 (0.5692)	3.1503 (0.0019)
Cumulative effect	1238.4424 (0.0679)	15389.7213 (0.8437)	−407.0998 (−0.0223)	1314.1911 (0.072)	706.4814 (0.0387)

Unit: ten thousand tons.

Note: data in the parentheses denote effect contribution of each factor.

**Table 3:** Effects of different factors of carbon emissions from energy consumption of Shandong province from 1996 to 2012 by Shapley value model.

Year	Population	Per capita GDP	Industrial structure	Energy consumption intensity	Energy consumption structure
1996	18.6577 (0.1035)	544.7407 (3.0233)	−17.0525 (−0.0946)	−914.0294 (−5.0728)	187.5007 (1.0406)
1997	25.9293 (2.123)	202.9057 (16.613)	59.0847 (4.8376)	−366.0543 (−29.9708)	90.3483 (7.3973)
1998	30.9145 (0.0504)	495.6131 (0.808)	24.2287 (0.0395)	218.1564 (0.3557)	−155.5511 (−0.2536)
1999	28.1396 (0.1791)	672.0179 (4.2778)	−4.8905 (-0.0311)	−626.9532 (−3.9909)	88.7799 (0.5651)
2000	71.6583 (11.306)	648.8608 (102.3755)	95.146 (15.0119)	−903.1046 (−142.4894)	81.1014 (12.796)
2001	30.8817 (0.0205)	516.9244 (0.3431)	−40.039 (−0.0266)	640.6682 (0.4252)	358.2127 (0.2378)
2002	31.6073 (0.1185)	723.4283 (2.7118)	74.862 (0.2806)	−848.6937 (−3.1814)	−247.9752 (−0.9295)
2003	33.9285 (0.0517)	865.3264 (1.3174)	290.8793 (0.4429)	−408.7098 (−0.6222)	−124.5917 (−0.1897)
2004	49.8875 (0.0302)	832.7956 (0.504)	279.4521 (0.1691)	347.5003 (0.2103)	142.8168 (0.0864)
2005	82.8111 (0.02)	634.6065 (0.153)	87.3402 (0.0211)	3850.5752 (0.9281)	−506.2825 (−0.122)
2006	92.2087 (0.0626)	971.3506 (0.6597)	60.0513 (0.0408)	224.413 (0.1524)	124.2799 (0.0844)
2007	97.3175 (0.0537)	1374.15 (0.7577)	−103.5861 (−0.0571)	177.2866 (0.0978)	268.2936 (0.1479)
2008	91.1412 (0.0852)	1331.4664 (1.2452)	−3.2492 (−0.003)	−470.7521 (−0.4402)	120.6902 (0.1129)
2009	102.3997 (0.0868)	738.3576 (0.6258)	−199.3902 (−0.169)	643.6489 (0.5455)	−105.1847 (−0.0892)
2010	223.6508 (0.163)	2503.4203 (1.8241)	−317.3014 (−0.2312)	−1028.4055 (−0.7494)	−8.9739 (−0.0065)
2011	126.0607 (0.0933)	1436.9178 (1.0631)	−294.2589 (−0.2177)	−163.7351 (−0.1211)	246.6197 (0.1825)
2012	111.2845 (0.0659)	1012.5553 (0.5997)	−396.3759 (−0.2348)	958.3766 (0.5676)	2.6483 (0.0016)
Cumulative effect	1248.4783 (0.0684)	15505.4374 (0.85)	−405.0995 (−0.0222)	1330.1876 (0.0729)	562.7325 (0.0308)

Unit: ten thousand tons.

Note: data in the parentheses denote effect contribution of each factor.

**Table 4:** Effects of different factors of carbon emissions from energy consumption of Shandong province from 1996 to 2012 by MRCI model.

Year	Population	Per capita GDP	Industrial structure	Energy consumption intensity	Energy consumption structure
1996	18.7097 (0.1038)	545.532 (3.0277)	−16.8651 (−0.0936)	−916.9586 (−5.089)	189.3992 (1.0511)
1997	25.9871 (2.1277)	203.33 (16.6477)	59.2243 (4.849)	−366.6415 (−30.0189)	90.3138 (7.3945)
1998	31.1165 (0.0507)	498.9151 (0.8134)	24.5535 (0.04)	220.3972 (0.3593)	−161.6207 (−0.2635)
1999	28.1391 (0.1791)	671.3708 (4.2737)	−5.5407 (−0.0353)	−623.095 (−3.9664)	86.2196 (0.5488)
2000	71.3501 (11.2574)	645.3571 (101.8227)	94.958 (14.9822)	−896.7928 (−141.4935)	78.7895 (12.4312)
2001	30.8292 (0.0205)	516.5466 (0.3428)	−39.7094 (−0.0264)	641.1385 (0.4255)	357.843 (0.2375)
2002	31.5713 (0.1183)	721.7347 (2.7054)	74.7581 (0.2802)	−848.244 (−3.1797)	−246.5914 (−0.9244)
2003	33.7607 (0.0514)	860.7699 (1.3105)	291.5284 (0.4438)	−415.7839 (−0.633)	−113.4424 (−0.1727)
2004	49.5702 (0.03)	828.1029 (0.5011)	280.6039 (0.1698)	383.6777 (0.2322)	110.4976 (0.0669)
2005	83.8507 (0.0202)	643.3808 (0.1551)	71.6141 (0.0173)	3986.9018 (0.9609)	−636.6968 (−0.1535)
2006	92.0984 (0.0626)	970.3362 (0.6591)	59.979 (0.0407)	224.2639 (0.1523)	125.626 (0.0853)
2007	97.373 (0.0537)	1375.1381 (0.7583)	−103.6562 (−0.0572)	177.5362 (0.0979)	267.0705 (0.1473)
2008	91.3382 (0.0854)	1334.1783 (1.2477)	−3.2638 (−0.0031)	−471.8053 (−0.4412)	118.849 (0.1111)
2009	103.2747 (0.0875)	744.7072 (0.6312)	−193.6072 (−0.1641)	671.9289 (0.5695)	−146.4722 (−0.1241)
2010	223.5026 (0.1629)	2500.0787 (1.8217)	−317.2535 (−0.2312)	−1026.4602 (−0.7479)	−7.4773 (−0.0054)
2011	126.0491 (0.0933)	1436.7093 (1.063)	−294.2331 (−0.2177)	−163.5335 (−0.121)	246.6123 (0.1825)
2012	111.2601 (0.0659)	1012.4191 (0.5996)	−396.0589 (−0.2346)	958.1979 (0.5675)	2.6705 (0.0016)
Cumulative effect	1249.7807 (0.0685)	15508.6068 (0.8502)	−412.9686 (−0.0226)	1534.7273 (0.0841)	361.5901 (0.0198)

Unit: ten thousand tons.

Note: data in the parentheses denote effect contribution of each factor.

**Table 5:** Effects of different factors of carbon emissions from energy consumption of Shandong province from 1996 to 2012 by combination model.

Year	Population	Per capita GDP	Industrial structure	Energy consumption intensity	Energy consumption structure
1996	18.6171 (0.1033)	543.2565 (3.015)	−16.9443 (−0.094)	−912.3936 (−5.0637)	187.2814 (1.0394)
1997	25.894 (2.1201)	202.6206 (16.5896)	59.0491 (4.8347)	−365.439 (−29.9205)	90.0889 (7.3761)
1998	30.8789 (0.0503)	495.211 (0.8074)	24.2834 (0.0396)	218.3353 (0.356)	−155.347 (−0.2533)
1999	28.0979 (0.1789)	670.8723 (4.2705)	−5.1427 (−0.0327)	−624.4251 (−3.9749)	87.6913 (0.5582)
2000	71.1532 (11.2264)	644.0476 (101.6161)	94.4276 (14.8985)	−895.9746 (−141.3644)	80.0082 (12.6235)
2001	30.8112 (0.0205)	516.1733 (0.3426)	−39.9421 (−0.0265)	640.0107 (0.4248)	359.5948 (0.2387)
2002	31.5379 (0.1182)	721.4754 (2.7045)	74.729 (0.2801)	−846.9327 (−3.1748)	−247.5808 (−0.9281)
2003	33.6382 (0.0512)	858.0803 (1.3064)	289.6273 (0.4409)	−409.9999 (−0.6242)	−114.5131 (−0.1743)
2004	49.5705 (0.03)	828.1353 (0.5012)	279.5362 (0.1692)	362.7881 (0.2195)	132.4221 (0.0801)
2005	82.4993 (0.0199)	632.8004 (0.1525)	82.9241 (0.02)	3873.9283 (0.9337)	−523.1016 (−0.1261)
2006	92.0423 (0.0625)	969.8237 (0.6587)	59.9366 (0.0407)	224.0574 (0.1522)	126.4435 (0.0859)
2007	97.1116 (0.0536)	1371.6699 (0.7564)	−103.2546 (−0.0569)	176.7234 (0.0975)	271.2112 (0.1496)
2008	90.9482 (0.0851)	1328.7577 (1.2426)	−3.289 (−0.0031)	−470.5597 (−0.4401)	123.4392 (0.1154)
2009	102.3551 (0.0868)	738.0958 (0.6256)	−197.2879 (−0.1672)	653.2911 (0.5537)	−116.6228 (−0.0988)
2010	223.3075 (0.1627)	2499.5851 (1.8213)	−316.9305 (−0.2309)	−1026.1363 (−0.7477)	−7.4355 (−0.0054)
2011	125.968 (0.0932)	1435.9975 (1.0624)	−294.1296 (−0.2176)	−163.4339 (−0.1209)	247.2021 (0.1829)
2012	111.1361 (0.0658)	1011.3187 (0.5989)	−395.9818 (−0.2345)	959.1927 (0.5681)	2.823 (0.0017)
Cumulative effect	1245.5671 (0.0683)	15467.9211 (0.8479)	−408.3892 (−0.0224)	1393.0323 (0.0764)	543.6051 (0.0298)

Unit: ten thousand tons.

Note: data in the parentheses denote effect contribution of each factor.

**Table 6:** Bivariate-Pearson correlation coefficients.

	Carbon emissions	Population	Per capita GDP	GDP proportion of the Secondary industry	Energy consumption intensity	Ratio of high-carbon energy
Carbon emissions	1.0000					
Population	0.963**	1.0000				
Per capita GDP	0.978**	0.994**	1.0000			
GDP proportion of the secondary industry	0.644**	0.704**	0.665**	1.0000		
Energy consumption intensity	0.591**	0.374	0.420*	0.349	1.0000	
Ratio of high-carbon energy	0.381	0.438*	0.410*	0.168	0.062	1.0000

Note: **indicates a significant level of 0.01, *indicates a significant level of 0.05.
